# Birmingham-group IncP-1α plasmids revisited: RP4, RP1 and RK2 are identical and their remnants can be detected in environmental isolates

**DOI:** 10.1099/mgen.0.001381

**Published:** 2025-03-28

**Authors:** Vuong Van Hung Le, Zhuang Gong, Lorrie Maccario, Emma Bousquet, Boris Parra, Arnaud Dechesne, Søren J. Sørensen, Joseph Nesme

**Affiliations:** 1 Living Systems Institute, University of Exeter, Exeter, UK; 2 Faculty of Health and Life Sciences, University of Exeter, Exeter, UK; 3 Section of Microbiology, Department of Biology, University of Copenhagen, Copenhagen, Denmark; 4 Laboratorio de Investigación de Agentes Antibacterianos (LIAA), Departamento de Microbiología, Facultad de Ciencias Biológicas, Universidad de Concepción, Concepción, Chile; 5 Instituto de Ciencias Naturales, Facultad de Medicina Veterinaria y Agronomía, Universidad de las Américas, Concepción, Chile; 6 Department of Biotechnology and Biomedicine, Technical University of Denmark, Lyngby, Denmark

**Keywords:** Birmingham group plasmids, conjugative plasmids, IncP-1, plasmid remnants

## Abstract

RP4, RP1, RK2 and R68 were isolated from the multidrug-resistant bacterial wound isolates in 1969 in the Birmingham Accident Hospital, Birmingham, England, and collectively called Birmingham-group IncP-1*α* plasmids. These plasmids have been widely used as models to study different aspects of plasmid biology, develop genetic delivery systems and design plasmid vectors. Early studies showed that these plasmids conferred the same antibiotic resistance profile, had a similar size and were undistinguishable from each other using DNA heteroduplex electron microscopy and restriction endonuclease analyses. These observations have led to the widely held assumption that they are identical, although there has been no conclusive supporting evidence. In this work, we sequenced the plasmids RP1 and RP4 from our laboratory strain collection and compared these new sequences with the plasmids RP4 and RK2 assembled from a publicly available sequencing database, showing that the RP1, RP4 and RK2 plasmids are 60 095 bp in length and identical at the nucleotide resolution. Noteworthily, the plasmid sequence is highly conserved despite having been distributed to different labs over 50 years and propagated in different bacterial hosts, strengthening the previous observation that the bacterial host adapts to the RP4/RP1/RK2 plasmid rather than the opposite. In the updated RP4/RP1/RK2 sequence, we found a fusion gene, called *pecM-orf2*, that was formed putatively by a genetic deletion event. By searching for *pecM-orf2* in the National Center for Biotechnology Information database, we detected remnants of the RP4/RP1/RK2 plasmid that carry features of laboratory-engineered vectors in bacterial environmental isolates, either in their chromosome or as a plasmid. This suggests a leak of these plasmids from the laboratory into the environment, which may subsequently impact bacterial evolution and raises concerns about the biocontainment of engineered plasmids when being handled in laboratory settings.

## Data Summary

Sequencing data analysed in this work are available in the National Center for Biotechnology Information (NCBI) database under the BioProject PRJNA904677. The raw sequencing data for RP4 is SRX24151815; the raw sequencing data for RP1 is SRX24151816. The annotated sequences of RP4 and RP1 are available with accession numbers of PP591959.1 and PP591960.1, respectively. The genome assembly accession numbers in the NCBI GenBank are as follows: *Pseudomonas* sp. H2/RP4::Tn*6048*, DAWWKB000000000, and *Pseudomonas putida* KT2440/RP4, DAWWKC000000000. The genome assembly accession numbers in the European Nucleotide Archive are as follows: MG1655/RK2, GCA_964237535. The authors confirm that all supporting data and protocols have been provided within the article.

**Impact Statement**

RP4, RP1 and RK2, collectively referred to as the Birmingham-group IncP-1*α* plasmids, form one of the earliest reported groups of antibiotic-resistance conferring plasmids. These plasmids have been widely used as model systems to study different aspects of plasmid biology and the spread of antibiotic resistance genes and to develop genetic delivery systems and vectors in a wide range of bacterial hosts. Early studies have suggested that RP4, RP1 and RK2 are likely identical, but without conclusive evidence. Here, we solved the complete sequence of these plasmids that were carried by different bacterial hosts and concluded that they are identical at the nucleotide resolution. This conclusion has two important implications. Firstly, this adds confidence to the current practice that uses interchangeably the knowledge obtained from previous studies that used any of these plasmids as the model. Secondly, considering the bacterial host–plasmid interaction evolution, our findings strengthen the previous observation that the hosts adapt to the Birmingham-group IncP-1*α* plasmids, rather than the opposite, to mitigate the fitness cost. We also report the detection of laboratory-engineered RP4/RP1/RK2-derived plasmids in the genome of environmental bacterial isolates, suggesting a possible scenario that these plasmids have been leaked from the laboratory into the environment. This raises concern about the imperfect biocontainment of man-made plasmids when being handled in laboratory settings.

## Introduction

The Birmingham-group IncP-1*α* conjugative plasmids include RP1, RP4, RK2 and R68, which were identified in multidrug-resistant bacterial isolates from the Medical Research Council Industrial Injuries and Burns Research Unit at the Birmingham Accident Hospital, Birmingham, England, in 1969 [1,2]. Particularly, the RP1 (synonymous with R18 and R1822) and R68 (synonymous with R6886) plasmids were isolated from the burn wound isolate *Pseudomonas aeruginosa* 1822 and 6886, respectively, while the RK2 plasmid was isolated from *Klebsiella aerogenes* K8841 [2,7]. The original source of the RP4 plasmid is less certain. This plasmid was first propagated and characterized in *Escherichia coli* J53 in Datta *et al.* (1971) before being distributed to different laboratories around the world [8], but it might have been RP1 due to a labelling error at some stages during the transfer and handling of the bacterial strain before reaching Datta’s laboratory [9]. Given that these plasmids are some of the earliest reported antibiotic-resistance conferring plasmids (also known as R factors), they have been widely used as model systems to study different aspects of plasmid biology (e.g. plasmid replication, maintenance, conjugation and plasmid–host interaction) and the spread of antimicrobial resistance determinants. They have also been employed to develop genetic delivery systems and design plasmid vectors in a broad range of bacterial hosts, including non-model bacteria.

Early studies showed that these plasmids have similar physical property and size and confer resistance to the same panel of antibiotics, including tetracycline, neomycin/kanamycin and carbenicillin [4,5, 9]. Pairwise comparisons using methods available at the time, including DNA heteroduplex electron microscopy [10] and restriction endonuclease analyses [11], concluded that these plasmids were indistinguishable from each other. With these observations, the Birmingham-group IncP-1*α* plasmids were often referred to as identical, although no conclusive evidence supports that statement. In 1994, the first complete sequence of the Birmingham-group IncP-1*α* plasmids was achieved by compiling and assembling the sequences of small DNA fragments derived from RP1, RP4, RK2 and R68, which were separately solved and reported in multiple previous studies [1]. While this work has provided a very useful reference sequence of a historically important plasmid group, there were caveats. Firstly, it is uncertain whether the Birmingham-group IncP-1*α* plasmids are identical at the nucleotide resolution given that the plasmid comparison methods mentioned above (i.e. DNA heteroduplex electron microscopy and restriction endonuclease analyses) were unable to detect small genetic differences (e.g. nucleotide substitutions and small deletions/insertions). At least, it is known that the R68 plasmid has some phenotypic differences from the others, such as sensitivity to plasmid-dependent phages (Pf3, PRR1) and a distinct interaction with the B3 prophage in recipient cells [12], suggesting that there must be some genetic differences between R68 and the others. Secondly, the relatively low accuracy of the first-generation sequencing (Sanger and Maxim–Gilbert) at the time, followed by the scaffolding of input sequences from different studies, may have introduced errors in the assembled plasmid sequence. This observation was emphasized in a later study when a small region of the RK2 plasmid was re-sequenced, revealing multiple nucleotide mismatches in comparison with the old reference sequence [13]. This urges a need for a re-sequencing campaign for the Birmingham-group IncP-1*α* plasmids.

In this work, we sequenced and assembled the RP1 and RP4 plasmids available from our laboratory as well as assembled the RP4 and RK2 plasmids from other laboratories using publicly available raw sequencing data. We establish that these plasmids are identical at the nucleotide resolution and generate a new high-quality annotated reference. In addition, we reported and used a gene fusion event, called *pecM-orf2*, to trace the presence of the Birmingham-group IncP-1*α* plasmid sequences in bacterial genomes deposited in the National Center for Biotechnology Information (NCBI) GenBank database. From this, some RP4/RP1/RK2 plasmid remnants and their derived synthetic plasmids were detected in the genome of environmental bacterial isolates, raising the question about their impact on the physiology and evolution of the corresponding bacterial hosts in nature.

## Methods

### Bacterial strains and plasmids

Bacterial strains used in this study are as follows: *E. coli* J53/RP4 (*E. coli* K12 (F^−^ *met pro*)/RP4) from [8] and *P. aeruginosa* PAO1/RP1, a kind gift from Professor Udo Bläsi, University of Vienna [14]. Bacteria were cultivated in Luria–Bertani (LB) broth at the shaking of 250 rotations per minute or LB agar (1.5% agar) at 37 °C unless stated otherwise. Tetracycline at 10 μg ml^−1^ was added to the media to maintain the RP4/RP1 plasmids.

### Plasmid extraction, sequencing and assembly

The RP4 and RP1 plasmids were extracted from 20 ml of the overnight culture of *E. coli* J53/RP4 and *P. aeruginosa* PAO1/RP1, respectively, using the Plasmid Mini AX kit (A&A Biotechnology) according to the manufacturer’s instructions. The DNA library was prepared using the Nextera XT DNA Library Preparation Kit, indexed with the Nextera XT Index Kit v2 Set A and sequenced on the Illumina 2×250 base paired-end v2 MiSeq platform. For data preprocessing, the adapters and the low-quality bases (the average quality score of a 4-base window <20) were trimmed from the raw reads, and the resulting trimmed reads that were shorter than 36 bases were then removed from the sequencing data using Trimmomatic v0.39 [15]. The resulting high-quality reads were assembled using Unicycler v0.5.0 with default parameters [16], and the genome assembly graph was visualized using Bandage v0.8.1 [17]. This workflow resulted in a single circular contig for RP1 and RP4 at 60 095 bp (Data S1 and S2). The read coverage depth was estimated to be 1495× for RP4 and 1410× for RP1. The sequence reads were then mapped back to the RP1 or RP4 assembly using Bowtie2 v2.5.0 at the *--very-sensitive* mode [18]. It showed that 96.87% of the RP4 sequencing reads were mapped to the RP4 assembly, while 96.63% of the RP1 sequencing reads were mapped to the RP1 assembly, indicating very low contamination of reads from bacterial chromosome. To confirm the host of each plasmid, the low-coverage-depth contigs were extracted from the intermediate assembly file of RP1 and RP4 (Data S3 and S4) and searched against the NCBI nucleotide sequence database using the megaBlast v2.15.0+ webserver with default parameters [19]. The contigs from the RP1 assembly were found to match the *P. aeruginosa* chromosome, and the contigs from the RP4 assembly were found to match to *E. coli* chromosome, confirming that these plasmid samples were extracted from the correct bacterial hosts. Our newly assembled RP1 and RP4 plasmids were annotated using Prokka v1.14.6 using the annotated IncP-1*α* Birmingham plasmid file as the reference (accession no. BN000925.1) [13,20] and visualized using Geneious v2024.0.4.

The same bioinformatic workflow was applied to recover the IncP-1*α* plasmids from the publicly available sequencing data: (i) RP4 propagated in *Pseudomonas* sp. H2 (accession no. SRR4444896), (ii) RP4 propagated in *Pseudomonas putida* KT2440 (accession no. SRR11267622) and (iii) RK2 propagated in *E. coli* MG1655 (accession no. ERR1957960). The assembled RP4, RP1 and RK2 plasmids and other IncP-1 plasmids were aligned using the progressiveMauve algorithm [21] and visualized using the Mauve software v20150226 [22]. The IncP-1 plasmids included in this analysis were pG527 (JX469830.1), pBS228 (AM261760.1), pWEC911 (JX469833.1), pTB11 (AJ744860.1), pB11 (CP002152.1), pB5 (CP002151.1), pSP21 (CP002153.1), pMNCN061 (LC623900.1), pMNCN064 (LC623901.1), pSN1104-59 (AP018709.1), pMNCI060 (LC623896.1), pBP136 (AB237782.1), pSM0227-07 (LC623903.1), pMNCI063 (LC623898.1), pYKBU009 (LC623927.1), pMH0621-02Tc (LC623884.1), pYKCT011-1 (LC623931.1), pYKAZ004 (LC623913.1), R751 (U67194.4), pB8 (AJ863570.1), pYKBF005 (LC623914.1), pKS208 (JQ432564.1), pAKD4 (GQ983559.1), pKJK5 (AM261282.1), pMCBF1 (AY950444.1), pDS1 (KC170283.1), p7ME01 (CP006601.1) and pYKCG107 (LC623928.1).

### Protein structure modelling

The structures of PecM_pTB11_ (accession no. CAG30851.1), Orf2_pTB11_ (accession no. CAG30856.1) and PecM-Orf2_RP4/RP1/RK2_ were modelled using ColabFold v1.3.0 with default parameters [23]. The modelled protein structures were visualized using ChimeraX v1.7 [24].

### Searching plasmids and plasmid remnants from the NCBI database

Retrieval of plasmids and plasmid remnants closely related to the Birmingham-group IncP-1*α* plasmids was done based on the presence of the fusion gene *pecM-orf2*. To this end, the PecM-Orf2 aa sequence extracted from the annotated RP4 plasmid was queried against the NCBI non-redundant protein sequence database using the blastp v2.15.0+ webserver with default parameters [25]. Also, the *pecM-orf2* nucleotide coding sequence was queried against the NCBI nucleotide sequence database using megaBlast v2.15.0+ webserver with default parameters [19]. The hits that apparently correspond to laboratory synthetic vectors (i.e. known synthetic vector names) were filtered out, while the ones found in natural bacterial isolates were retained for further analysis. The contigs that contained the *pecM-orf2* sequence were compared to the RP4 sequence and visualized using the megaBlast algorithm of the blastn package v2.13.0 through the sequenceserver platform v2.0.0 [19,26]. The region of the hit contigs matching the RP4 sequence was queried against the NCBI nucleotide database using megaBlast v2.15.0+ webserver with default parameters [19] to identify the source of the hit contigs, which were then visualized using the blast Ring Image Generator (BRIG) software v0.95 [27].

## Results and discussion

### The RP4, RP1 and RK2 plasmids are identical at the nucleotide resolution

**Fig. 1.**
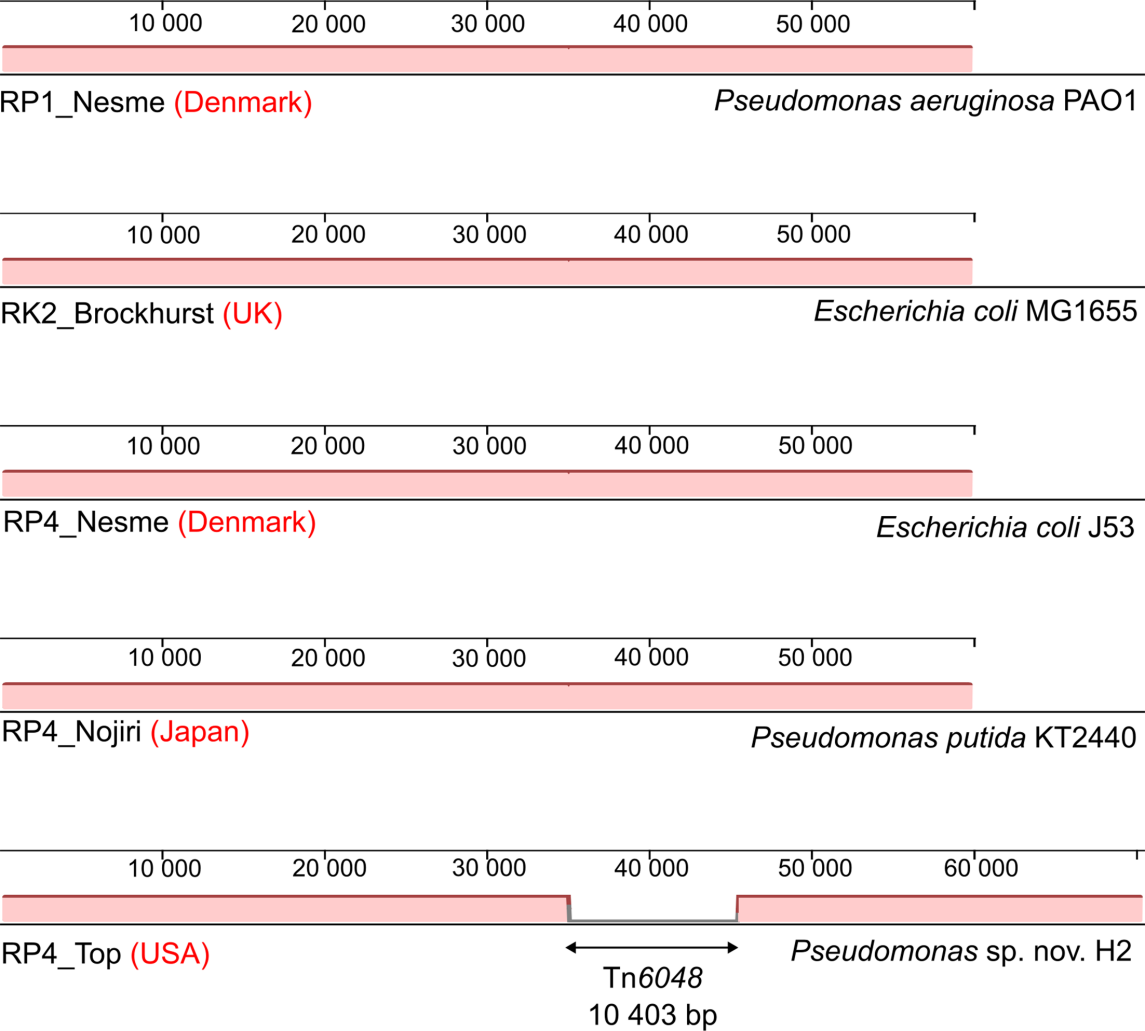
Alignment of the Birmingham-group IncP-1*α* plasmids. The text next to the plasmid name indicates the laboratory where the plasmid sequencing was done and the country of origin (in red). The right text is the bacterial host that was used to propagate the plasmid. The plasmids were aligned using the progressiveMauve algorithm on the Mauve software. Locally collinear blocks are colour coded. Sequence orientation relative to the reference genome (RP1_Nesme): above the centre line, forward orientation; below the centre line, reverse orientation. Double-headed arrow, insertion of Tn*6048*.

To assemble the complete sequence of the Birmingham-group IncP-1*α* plasmid, we started with the two strains available in our lab bacterial collection: *E. coli* J53/RP4 and *P. aeruginosa* PAO1/RP1. The RP4 and RP1 plasmids were propagated in their corresponding host, extracted and sequenced using Illumina short-read sequencing. Assembling the sequencing reads of each plasmid resulted in a single circular contig of 60 095 bp (Fig. 1). We also retrieved the publicly available whole-genome sequencing data of bacterial strains (two different *Pseudomonas* species and one *E. coli* strain) containing RK2 or RP4 from three bacterial host–plasmid interaction evolutionary studies and were able to assemble and extract a circular contig corresponding to a conjugative plasmid from each dataset [28,30]. These complete plasmid sequences are 100% identical at the nucleotide resolution over the same length of 60 095 bp (Fig. 1). The absence of any mismatch is striking given that these plasmids have been distributed to different laboratories around the world for over 50 years and propagated in different bacterial strains (Fig. 1). Particularly, our RP1 and RP4 plasmids were propagated in *P. aeruginosa* PAO1 and *E. coli* J53, respectively. The RP4 plasmids from the Nojiri lab and the Top lab were propagated in *P. putida* KT2440 and *Pseudomonas* sp. nov. H2, respectively [29,30], while RK2 from the Brockhurst lab was grown in the *E. coli* laboratory strain MG1655 [28]. Interestingly, similar observations have also been reported in other plasmid groups. For example, pRK100, a conjugative 142-kb-long multireplicon plasmid identified from a clinical strain of *E. coli* KS533 in Slovenia (1990), was found to share extremely high sequence identity (>99.95%) with the homologous plasmids that were isolated more than 20 years later in Australia and the UK [31]. The plasmids pZM3 and pRSB225 were isolated from Algeria and Germany, respectively, 43 years apart, but shared 99.97% identity across 96% of the backbone [32]. Also, plasmids carried by sequence type 121 *Listeria monocytogenes* strains isolated from different food production plants across Europe (i.e. Austria, Ireland, Denmark, Italy and Spain) from 2000 to 2014 were found almost identical (>99.9% identity) to each other [33].

On top of that, when conducting a plasmid–host adaptive evolution between *Pseudomonas* sp. nov. H2 and RP4 for 600 generations, Loftie-Eaton *et al.* showed that the fitness cost imposed by the plasmid carriage was compensated exclusively by chromosomal mutations in two accessory-helicase-encoding genes (*uvrD* and *xpd*/*rad3*-like) and the RNA polymerase *β*-subunit-encoding gene *rpoB*, whereas the plasmid sequence remained unchanged [29]. Similarly, Kawano *et al.* reported that the mutations alleviating the RP4-associated fitness cost in *P. putida* KT2440 were located on the chromosomal intergenic region encoding a small RNA, called Pit174 [30]. The mutations in this chromosomal locus decreased the transcription of the Pit174 sRNA, whose overexpression upon RP4 carriage was found to negatively affect the host fitness [30]. In the adaptive evolution experiments between *E. coli* MG1655 and RK2 conducted in either antibiotic-free, tetracycline-only-containing, ampicillin-only-containing, tetracycline/ampicillin-co-present or tetracycline/ampicillin cycling media reported by Bottery *et al.*, all the compensatory mutations were found exclusively in the chromosome in tetracycline-free conditions such as in the Na^+^:H^+^ antiporter-encoding *nhaA* and *higA* of the *higAB* toxin–antitoxin system. Only in tetracycline-containing conditions, compensatory mutations occurred both in the chromosome and the plasmid [28,34]. Taken together, these findings from previous literature and the identical sequence of Birmingham-group IncP-1*α* plasmids reported in this study support the notion that the bacterial hosts tend to adapt to the RP4/RP1/RK2 plasmids via chromosomal mutations to reduce the fitness cost of plasmid carriage, rather than the opposite.

There was one noticeable exception that the RP4 plasmid from the Top lab had an insertion of Tn*6048* (10 403 bp) in the intergenic region between *parB* and *parC* with a 5 bp duplication [29]. Notably, the plasmids pAKD16 (IncP-1*ε* group) and pMOL98 (PromA group) reported by the same lab also had an identical Tn*6048*, which was considered to be obtained from the genome of the recipient strain AE815, a plasmid-cured derivative of the *Cupriavidus metallidurans* strain CH34, during triparental mating plasmid isolation [35,36]. We speculate that RP4 used in Loftie-Eaton *et al.* [29] had captured Tn*6048* at some point when it was propagated in a Tn*6048*-containing host, likely the same strain AE815 [37]. While it is unknown when RP4 had first captured Tn*6048*, we propose that the RP4 plasmid distributed from Loftie-Eaton *et al.* [29] should be renamed to RP4::Tn*6048* to reflect its sequence.

### Correcting the old record of the Birmingham-group IncP-1*α* plasmids

**Fig. 2.**
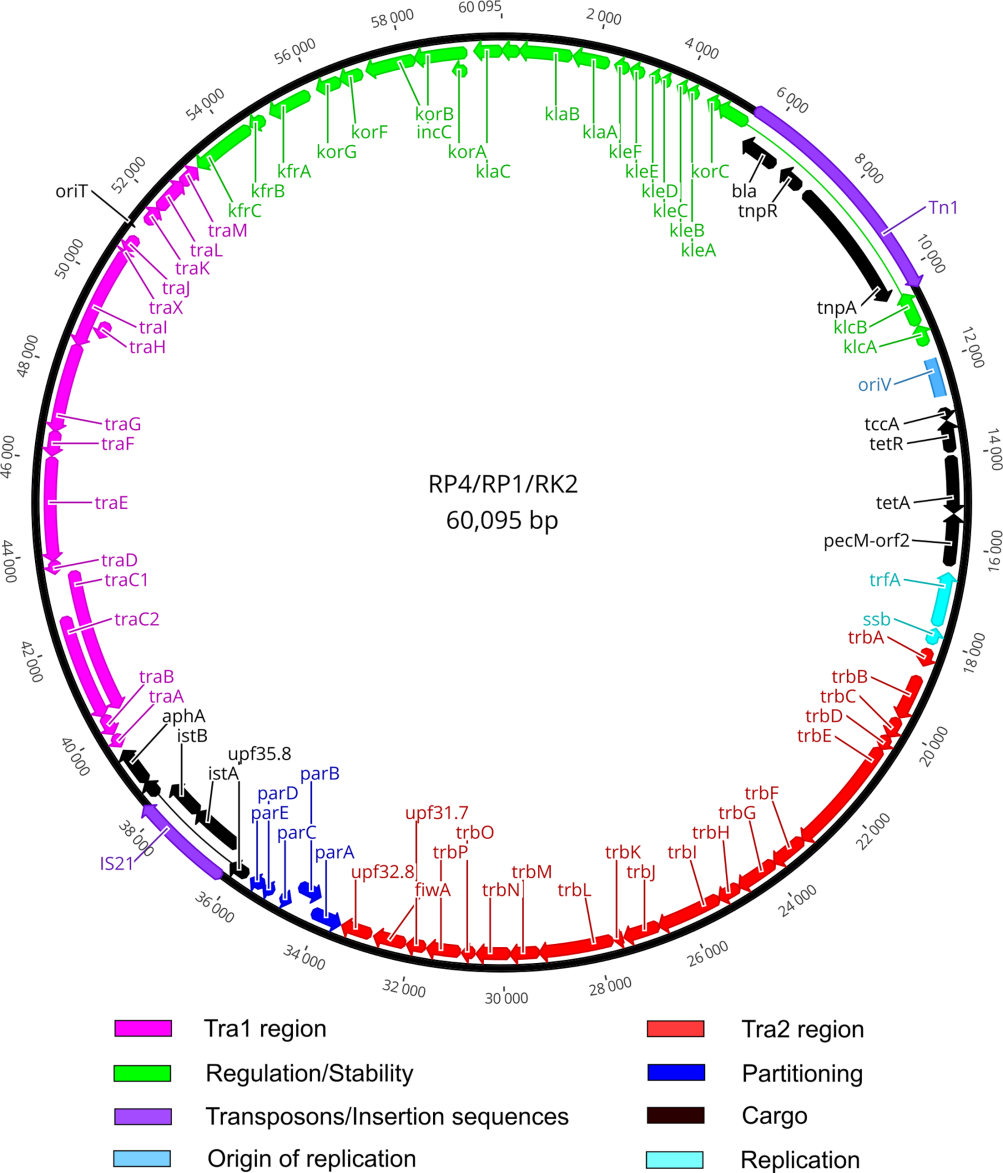
Genetic map of the RP1/RP4/RK2 plasmids. Genes are colour coded by their functions.

The first complete sequence of a representative Birmingham-group IncP-1*α* plasmid, consisting of 60 099 bp, was reported in 1994 (GenBank: L27758.1) and achieved by compiling fragmented sequences of the RP1, RP4, RK2 and R68 plasmids [1]. In 2007, the *kfrA-C* region of the RK2 plasmid was re-sequenced, identifying five mismatches in comparison with the original record, and therefore, a new 60‌ 096 bp Birmingham-group IncP-1*α* annotated sequence was then updated (GenBank: BN000925.1) [13]. Nonetheless, errors in the rest of this reference sequence were inevitable due to the low accuracy of the first-generation sequencing (i.e. Sanger and Maxam–Gilbert sequencing) at the time and the method of assembly by compiling different plasmids. To examine that, we compared our newly assembled RP4/RP1/RK2 sequence with the existing Birmingham IncP-1*α* record (BN000925.1), reporting 36 mismatches, many of which are expected to cause changes in the protein aa sequence, such as the aminoglycoside resistance gene *aphA* and the Tn*1* transposase *tnpA*, or changes in the reading frame as seen in *upf16.5* and *korF* (Table 1). It is worth noting that these mismatches have previously been noted when comparing other IncP-1*α* plasmids (pTB11, pBS228, pB5, pB11 and pSP21) with the BN000925.1 reference sequence [13,38, 39]. With the newly assembled RP4/RP1/RK2 sequence, we concluded that these reported differences were indeed artefacts due to occasional sequencing errors in the BN000925.1 sequence record. The updated sequence of the Birmingham-group IncP-1*α* plasmids is now available in the NCBI GenBank database (PP591959.1 for RP4 and PP591960.1 for RP1) (Fig. 2). The findings here also suggest an important implication that older plasmid data need to be re-checked, re-sequenced and updated when newer/better methods become available.

**Table 1.** Mismatches between the newly assembled RP4/RP1/RK2 sequence and the existing Birmingham IncP-1*α* sequence record (GenBank: BN000925.1)

nt mismatch*	Locus	Effect on annotation
8770InsC, 8773DelA, A9791G, G9802A, A10089C, T10359A	*tnpA*	Ala457Arg, Asp797Gly, Asp801Asn
12240InsA, T12406G, A12688T	Intergenic: *klcA*−*tetR*	no
15560DelT, C15639G, 15896InsC, 16411InsT	*upf16.5*	Translational fusion of *pecM-orf2*
C31028G	*trbP*	Pro35Ala
31939DelC, 31942InsG	*upf31.7*	Pro87Arg, Pro88Ala
A36655G	*istA*	Arg116Gly
38387InsCG, 38390DelT, C38393T, 38420DelC, 38508DelG, 38525DelC, 39196DelG, 39146DelG, 39179InsGC	*aphA*	VQQWTTHAGLPERGS[127-141] FNSDHACRLARARER
53067InsT	Intergenic: *traM−kfrC*	no
54833DelC	Intergenic: *kfrA−kfrB*	no
56817DelG	*korF*	Frameshift after Gly99

* The coordinates are based on the existing reference BN000925.1. Ins, insertion; Del, deletion; no, no effect on any protein sequence.

### High sequence conservation of the IncP-1*α* plasmid group

**Fig. 3.**
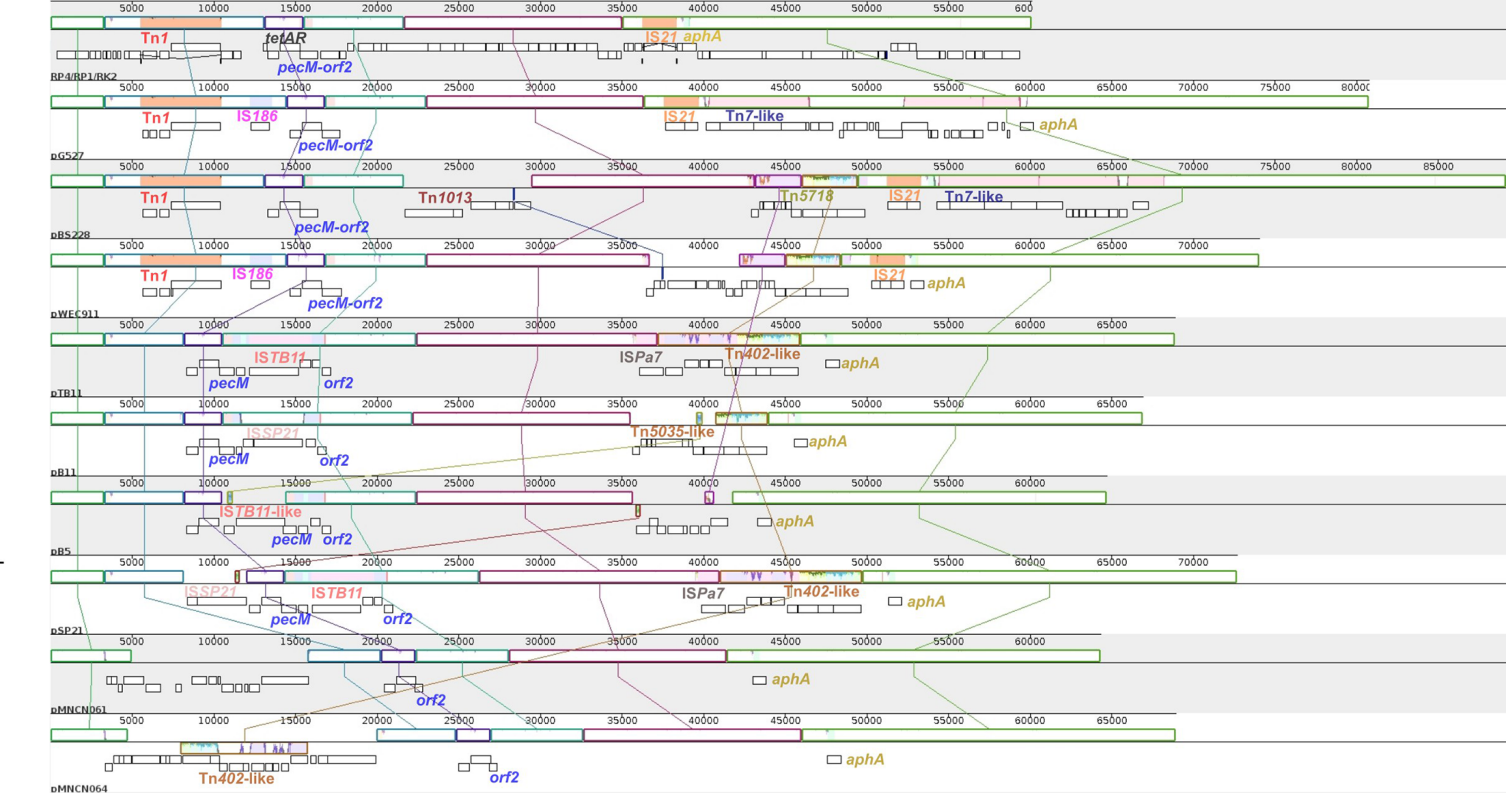
Alignment of the IncP-1*α* plasmid sequences. The plasmids were aligned using the progressiveMauve algorithm on the Mauve software. Locally collinear blocks are colour coded. Sequence orientation relative to the reference genome (RP4/RP1/RK2): above the centre line, forward orientation; below the centre line, reverse orientation. Except RP4/RP1/RK2, most of the gene annotations of the other plasmids (white boxes under locally collinear blocks) were removed for clarity purposes. The GenBank accession numbers for the IncP-1*α* plasmids are as follows: pG527 (JX469830.1), pBS228 (AM261760.1), pWEC911 (JX469833.1), pTB11 (AJ744860.1), pB11 (CP002152.1), pB5 (CP002151.1), pSP21 (CP002153.1), pMNCN061 (LC623900.1) and pMNCN064 (LC623901.1). There were 99 SNPs along the common aligned region of 47 981 bp across the IncP-1*α* plasmids under study.

Given the high conservation of the Birmingham-group IncP-1*α* plasmids, we expanded the sequence conservation analysis to all other published plasmids of the IncP-1*α* incompatibility group. It is worth mentioning that Szczepanowski *et al.* (2011) conducted such a comparative analysis with the IncP-1*α* plasmids available at the time, including the Birmingham-group plasmid representative (BN000925.1), pTB11, pBS228, pB5, pB11 and pSP21, and found that the plasmid sequence was highly conserved, with more than 99.9% identity [39]. In the present analysis, we included IncP-1*α* plasmids that have been isolated and sequenced since then, including pG527, pWEC911, pMNCN061 and pMNCN064 and the corrected sequence of RP4/RP1/RK2. Interestingly, their DNA sequences were still highly conserved. There were only 99 SNPs across the common aligned DNA length of 47 981 bp, which was equivalent to 99.79% identity (Fig. 3). By contrast, when the plasmids from another IncP-1 subgroup (i.e. *β*-1) were aligned, the sequence identity was decreased to 92.57% over the aligned region of 35 196 bp (Fig. S1). We also compared the representative of each IncP-1 subgroup with the RP4/RP1/RK2 plasmid to examine the divergence between the subgroups. As shown in Fig. S2a–h, the pairwise identity between the RP4/RP1/RK2 plasmid and other IncP-1 subgroups was rather low, ranging from 62.53 to 77.64%, whereas the IncP-1*α* within-subgroup alignment between the RP4/RP1/RK2 plasmid and pTB11 was also shown, as a contrasting example, with the identity being up to 99.97% (Fig. S2i). Overall, this indicates that the sequence of the IncP-1*α* subgroup is highly conserved in a very diverse IncP-1 incompatibility group, and this conservation is not a general feature for all IncP-1 subgroups.

We suggest several hypotheses that could explain the very high conservation of the IncP-1*α* subgroup. Firstly, in the plasmid–host interaction evolution to mitigate the plasmid-borne burden, the plasmid provides a much smaller target for mutations than the chromosome. For example, an IncP-1*α* plasmid at a length of ~65 kb with a copy number of 5 would have a total of 325 kb for mutations, which is ~14-fold smaller than a typical *E. coli* chromosome of 4.6 Mb. Secondly, a mutation occurring on one copy of the multicopy plasmid without being strongly selected is more likely to be lost from the population due to plasmid segregational drift [40]. Thirdly, a mechanism called gene conversion, which involves non-reciprocal transfer of information between homologous loci, may play a role in conserving the sequence of plasmids that exist in multiple copies in the bacterial cell. Particularly, when a mutation occurs on one plasmid copy, this mutant allele can be converted back to the original version by other non-mutated plasmid templates, probably via homologous recombination [41,42].

By contrast, there are factors that can drive the divergence of the plasmid sequence, as observed in the IncP-1 *β*-1 subgroup, in comparison with the IncP-1*α* subgroup. For example, the sequence divergence of a plasmid subgroup could be dictated by the diversity of their evolutionary hosts via two possible mechanisms. Firstly, the plasmid DNA sequence can be ameliorated during the residence time within a particular host to match the host DNA *k*-mer signature pattern [43]. The more diverse bacterial hosts that a plasmid subgroup has spread into and evolved with, the more DNA signature pattern of different hosts would have been incorporated into the plasmid sequence and therefore the more divergence of the subgroup. The second mechanism is via variations in the copy number of the same plasmid in different hosts. For example, based on the read-depth-coverage ratio between the plasmid and chromosome, we estimated that RK2 exists in 4.47 copies in *E. coli* MG1655, RP4::Tn*6048* in 1.45 copies in *Pseudomonas* sp. H2 and RP4 in 3.16 copies in *P. putida* KT2442. A mutation in one copy of a high-copy-number plasmid is more likely to be lost from the population by segregational drift or gene conversion than that of a low-copy-number plasmid [40]. Another possible factor might be the level of control over the plasmid gene expression. A plasmid subgroup with tightly controlled regulation of its gene expression may be more constrained from mutations because any change might be deleterious; thus, they would have a lower evolution rate. This reasoning is conceivable, given that the IncP-1*α* plasmid replication, maintenance and conjugative transfer functions are strictly repressed by sophisticated regulatory circuits of at least four plasmid-encoded global regulators, KorA, KorB, TrbA and KorC [44,47]. Lastly, the selection pressure in the growth environment might play an important role in plasmid evolvability. As shown by the RK2 plasmid-*E. coli* evolution experiment by Bottery *et al.* [28], the presence of tetracycline in the medium selects for both chromosomal and plasmid mutations, while the tetracycline-free medium selects exclusively for chromosomal mutations.

### A gene fusion formed in the Birmingham-group IncP-1*α* plasmids

**Fig. 4.**
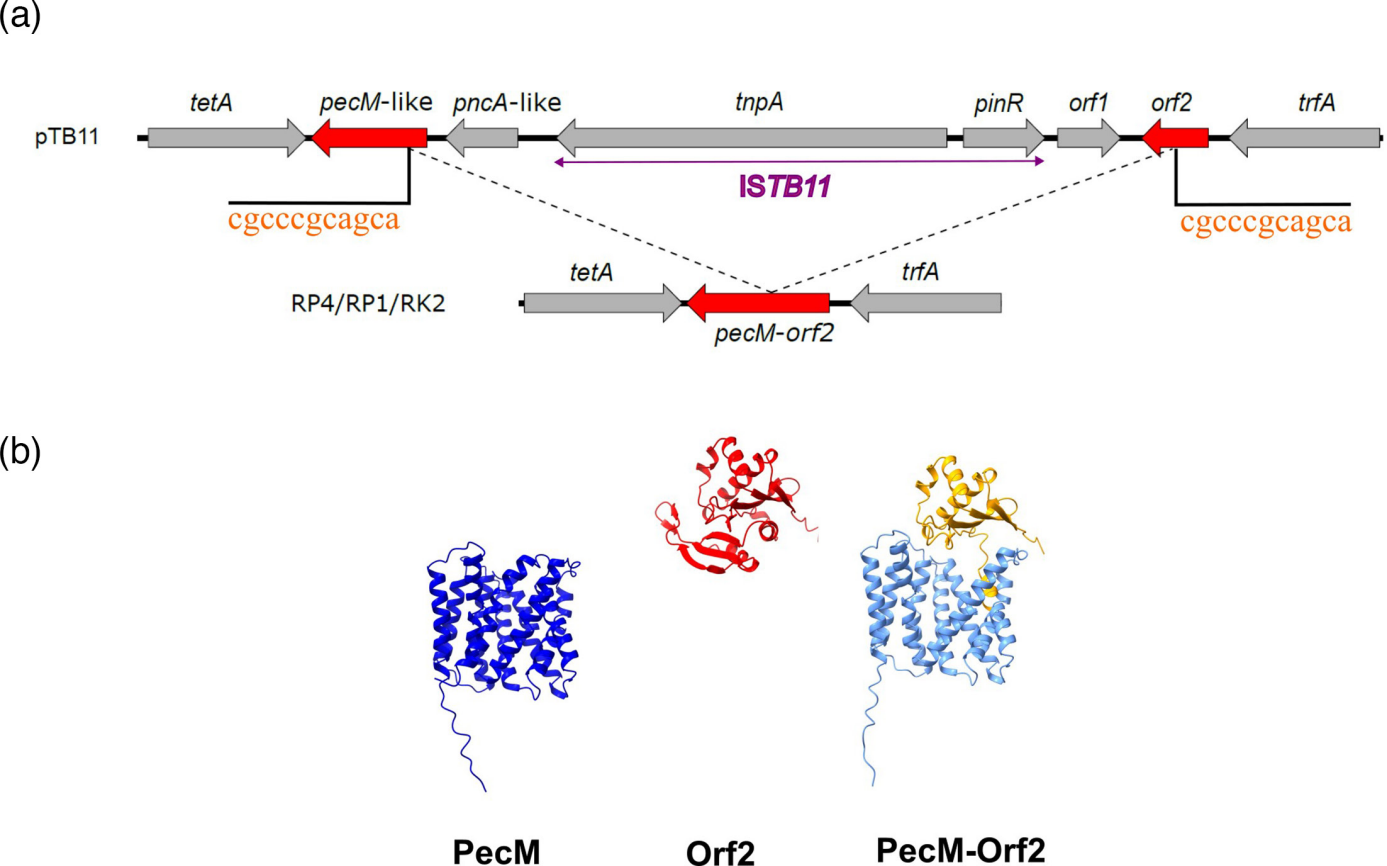
The putative fusion of the *pecM*_pTB11_ and *orf2*_pTB11_ in the Birmingham-group IncP-1*α* plasmid. (**a**) Comparative genetic maps in the *tetA-trfA* region of the RP4/RP1/RK2 and pTB11 plasmids. The dashed lines indicate a deletion putatively caused by a recombination of two 11-nt direct repeats (letters in orange), leading to the formation of the fusion gene *pecM-orf2*. The pTB11 plasmid has IS*TB11* containing the two genes: *tnpA* and *pinR*. (**b**) Cartoon representation of the structure of the PecM_pTB11_, Orf2_pTB11_ and PecM-Orf2_RP4/RP1/RK2_ modelled by ColabFold and visualized with ChimeraX. In the structure of PecM-Orf2_RP4/RP1/RK2_, the light blue region represents the residues originating from PecM_pTB11_, and the yellow region represents those originating from Orf2_pTB11_.

Among the mismatches between our newly assembled Birmingham**-**group IncP-1*α* plasmid and the existing reference sequence (BN000925.1), a noticeable observation is the cluster of single nucleotide insertions and deletions in the *upf16.5* region, causing a shift in the ORF (Table 1). The annotation of the *upf16.5* gene in the BN000925.1 reference was likely an artefact due to sequencing error since this protein version was also not found in any other plasmids when searching the NCBI database. We called the protein encoded by this locus in the newly assembled RP4/RP1/RK2 plasmid as PecM-Orf2, which is 204-residue longer than the protein encoded by the *upf16.5* gene due to earlier encounter of a stop codon in the *upf16.5* coding sequence. The naming of *pecM-orf2* was to reflect its likely origin in which the PecM-Orf2 protein is a fusion between the C-terminal portion of the PecM-like protein and the N-terminal portion of the Orf2 protein, whose intact encoding genes can be found in pTB11, pB11, pB5 and pSP21 (Fig. 3); those are other IncP-1*α* plasmids isolated from different wastewater treatment plant samples in Germany [38,39]. Meanwhile, the *pecM-orf2* gene was also present in pBS228 [13], pWEC911 and pG527 [48] (Fig. 3). The generation of the so-called *pecM-orf2* fusion has putatively been enabled by a recombination event of two 11-nt direct repeats within the *pecM*-like and *orf2* genes when comparing RP4/RP1/RK2 to pTB11 (Fig. 4a). It should be emphasized here that we use pTB11 as an example for illustration; the genesis of *pecM-orf2* could have occurred from any genetic arrangement in the region between *pecM* and *orf2* in pB11, pB5, pSP21 or other yet-to-be-isolated IncP-1*α* plasmids (Fig. 3). In some cases, there is an insertion sequence in this region, such as in pTB11, pSP21 and pB11 (Fig. 3), and it is unknown whether or not the *pecM-orf2* formation was facilitated by one of these insertion element (IS) elements.

The reverse scenario that an insertional event splits the *pecM-orf2* gene into two separate genes, *pecM* and *orf2*, is very unlikely for several reasons. Through structural modelling with ColabFold [23], PecM_pTB11_ and Orf2_pTB11_ were predicted to have a complete structural fold of a drug/metabolite transporter inner-membrane efflux pump and a GCN5-N-acetyltransferase, respectively (Fig. 4b). Meanwhile, the PecM-Orf2_RP4/RP1/RK2_ fusion protein was predicted to retain the fold of the constituting PecM_pTB11_ and Orf2_pTB11_ proteins yet missing the first transmembrane helix present in PecM_pTB11_ and the C-terminal region of Orf2_pTB11_. It is inconceivable for an insertional event to split *pecM-orf2* to perfectly restore two complete protein structures. As described in the next section, *pecM-orf2* homologues are rarely found in any bacteria or plasmids, suggesting that the formation of this protein is a rare and recent event.

### Tracing the *pecM-orf2* fusion gene detects remnant of Birmingham-group IncP-1*α* plasmids in bacterial environmental isolates

**Fig. 5.**
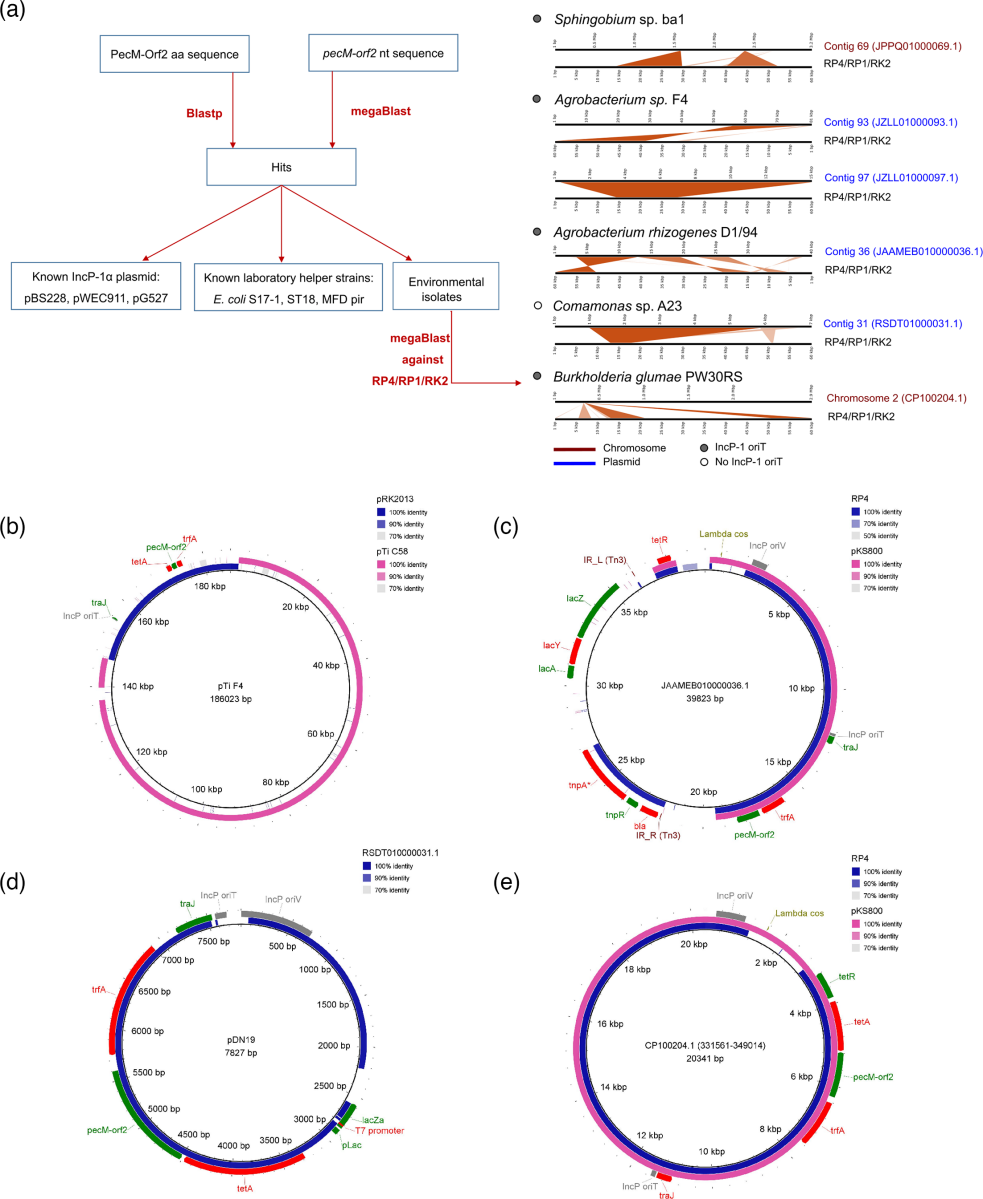
Detection of the remnants of the Birmingham-group IncP-1α plasmids in natural bacterial isolates. (**a**) Workflow to identify the distribution of the *pecM-orf2* fusion gene. The DNA region from bacterial environmental isolates matching the RP4/RP1/RK2 sequence was determined and visualized using megaBlast through the sequenceserver platform. Grey circle, presence of the IncP-1 *oriT* sequence; white circle, absence of the IncP-1 *oriT* sequence. Blue text, the hit contigs are plasmid-derived; orange text, the hit contigs are chromosome-derived. (**b**) Comparison of the pTi F4 plasmid from *Agrobacterium* sp. strain F4 with the RK2-derived plasmid RK2013 (OQ536579.1) and the tumour-inducing plasmid pTi C58 (AE007871.2). The pTi F4 plasmid was composed of three contigs (JZLL01000092.1, JZLL01000093.1 and JZLL01000097.1). (**c**) Comparison of the hit contig from *Agrobacterium rhizogenes* D1/94 with RP4 and the cosmid pKS800 (AB521037.1). (**d**) Comparison of the plasmid pDN19 (AF327711.1) with the hit contig from *Comamonas* sp. A23. (**e**) Comparison of the hit region of chromosome 2 of *Burkholderia glumae* PW30RS with RP4 and pKS800 (AB521037.1). The comparison and visualization were done with BRIG v0.95. The DNA sequence in the centre of the ring is set as a reference. The colour intensity of eccentric circles indicates the degree of sequence similarity.

Given the unique feature of the *pecM-orf2* fusion, we next searched for the distribution of this gene/protein in the NCBI database (Fig. 5a). After removing the hits that were laboratory synthetic vectors derived from RP4/RP1/RK2, we recovered other *pecM-orf2*-containing IncP-1*α* plasmids, including pBS228 [13], pWEC911 and pG527 [48], as already shown (Fig. 3), and the *E. coli* laboratory conjugation-helper strains with a Birmingham-group IncP-1*α* plasmid integrated into their chromosome [49]. Apart from that, we only found five hits from bacterial environmental isolates. This very limited prevalence of *pecM-orf2* indicates that the *pecM-orf2* fusion event may be a very rare and recent event that seems to be limited within IncP-1*α* plasmids (see the following section for the source of *pecM-orf2* in the environmental isolates).

We further analysed these environmental bacterial hits to trace the source of *pecM-orf2*. Comparing their whole genomes against the RP4/RP1/RK2 sequence showed that three hits are located on plasmid contigs and the other two are located on chromosomes (Fig. 5a, right panel). Among the chromosomal hits, *Sphingobium* sp. strain ba1, isolated in Italy [50], contains a total of ~27 kb of the RP4/RP1/RK2 plasmid in two loci of a chromosomal contig, indicating a historical entry of the plasmid followed by gene loss and chromosomal rearrangement. By contrast, *Burkholderia glumae* PW30RS, isolated in Korea [51], contains a remnant of the RP4/RP1/RK2 plasmid at a single locus on chromosome 2. Notably, the plasmid remnants on the chromosome of these two bacterial isolates carry the origin of transfer, *oriT*, making these chromosomes theoretically mobilizable in the presence of compatible conjugative machinery, which might facilitate horizontal chromosomal transfer and therefore impact bacterial evolution. Future research evaluating the chromosomal mobilizing ability of these strains is warranted.

Interestingly, except for *Sphingobium* sp. strain ba1, we were able to trace the source of the RP4/RP1/RK2 remnants that were found likely to derive from laboratory-engineered plasmids. Particularly, in *Agrobacterium* sp. strain F4 isolated from Germany, we found a plasmid, named pTi F4, following the authors’ original naming [52], which was composed of three contigs in the genome assembly (Fig. 5b). The pTi F4 is a cointegrate of two plasmids, including an RK2-derived plasmid pRK2013 and a T-DNA-disarmed tumour-inducing plasmid pTi C58. This design has been previously reported for delivery of a DNA cargo from *Agrobacterium* sp. into the plant cell [53]. Meanwhile, the plant isolate *Agrobacterium rhizogenes* D1/94 from the USA [54] contains a plasmid that resembles a fusion of the RK2-derived cosmid pKS800 and an engineered promoter-less Tn*3-lacZYA* (Fig. 5c). This design was used to study the gene expression under stress conditions at both transcriptional and translational levels [55,57]. *Comamonas* sp. A23 was isolated in cadmium-contaminated paddy soil in China [58]. We found that this isolate contains a small plasmid contig (~8 kb) that is almost identical to the broad-host-range RK2-derived vector pDN19, including signs of engineering, such as T7 promoter and *lacZ*-encoding sequence [59]. However, the plasmid in *Comamonas* sp. A23 had lost the *oriT* region. For the Korean isolate *B. glumae* PW30RS, the RP4/RP1/RK2 remnant in chromosome 2 is identical to the cosmid pKS800 [55].

Overall, these mentioned plasmids and plasmid remnants detected in environmental isolates from different countries carry features of synthetic plasmids made in the laboratories (e.g. lambda *cos* package site, T7 promoter, Tn*3-lacZYA*) and could suggest multiple leakage events from the laboratory to natural environments. However, it should be made clear here that our findings are solely based on literature reports from other scientists. There is no way to rule out contamination during the sequencing process and/or assembly errors as the source of the sequences. To be irrefutable, one would have to corroborate these findings with additional experimental work, for example, PCR amplification of a region containing synthetic plasmid remnants using primers binding on flanking regions of the identified synthetic plasmid remnants.

The presence of the IncP-1*α* plasmid remnants, including the *oriT* region, was previously noted in the chromosome of *P. aeruginosa* and *Shigella flexneri* by Chiu and Thomas [60]. Here, we expand the detection of the IncP-1*α* remnants to other bacterial genera, including *Sphingobium* and *Burkholderia*. Since these remnants indicate the past integration event of the plasmid into the chromosome, this provides a useful strategy to infer the host range of the plasmid, in an analogous manner to the determination of the phage host range using prophages and prophage remnants. It is worth noting that in this work, we found the IncP-1*α* plasmid and plasmid remnants in the NCBI database by querying the *pecM-orf2* fusion gene only, which is a very conservative approach. It is reasonable to speculate that the distribution of the conjugative plasmid remnants would be more widespread than currently known if other components of the plasmid (e.g. the *oriT* region) and other plasmid incompatibility groups (e.g. IncF, IncW and IncN) were included in the search. This leads to interesting questions about how these remnants affect bacterial evolution in nature and whether integrating the plasmid remnant into the chromosome is a common strategy used by the bacterial host to obtain beneficial traits without bearing the cost of maintaining the whole autonomously replicated plasmid.

### Conclusions and perspectives

The Birmingham-group IncP-1*α* plasmids (RP1, RP4 and RK2) are historically important multidrug-resistant plasmids that have been widely used as model systems to study different aspects of plasmid biology and dissemination of antibiotic resistance determinants since their discovery in 1969. In this work, we conclude that RP4, RP1 and RK2 are perfectly identical, adding high confidence to the current practice that uses interchangeably the knowledge accumulated from previous studies using any of these plasmids as their model over the past five decades.

With the updated RP4/RP1/RK2 sequence, we reported a fusion gene, namely, *pecM-orf2*, between *tetA* and *trfA*. It is tempting to characterize the function of the PecM-Orf2 protein in future work and examine if this possesses any emerging function different from its two constituting proteins, PecM_pTB11_ and Orf2_pTB11_, whose functions are also uncharacterized. While this was not the scope of this study, the detection of seemingly laboratory-engineered plasmids derived from RP4/RP1/RK2 in environmental bacterial isolates from different parts of the world suggests leakage events of these plasmids from research settings to natural environments, raising questions on what biocontainment measures should be implemented to minimize the number and the consequences of synthetic plasmid leakage events. It should be added here that the observations are based on literature and sequence databases and should be verified by orthogonal methods, such as PCR amplification or single-molecule long-read sequencing. Additionally, we only focused on a very restricted group of plasmids. Future research about these events should employ an exhaustive search with the inclusion of more plasmid markers, plasmid incompatibility groups and environmental metagenomic datasets.

## supplementary material

10.1099/mgen.0.001381 Uncited Table S1.

## Abbreviations

BRIG: blast ring image generator
IS: insertion element
LB: Luria–Bertani
NCBI: National Center for Biotechnology Information
